# Ethnic Disparities in Mental Health Among Adults in China

**DOI:** 10.1001/jamanetworkopen.2025.9591

**Published:** 2025-05-15

**Authors:** Yi Guo, Yibo Wu, Zhongjian Liu, Siyuan Fan, Haibo Wang

**Affiliations:** 1 Peking University First Hospital, Peking University, Beijing, China; 2 Clinical Research Institute, Institute of Advanced Clinical Medicine, Peking University, Beijing, China; 3 School of Public Health, Peking University, Beijing, China; 4 School of Public Health, Imperial College London, London, United Kingdom; 5 Key Laboratory of Epidemiology of Major Diseases (Peking University), Ministry of Education, Beijing, China

## Abstract

**Importance:**

There are significant gaps in the research on ethnic disparities in mental health status among adults nationwide in China. Evidence is needed to fill these gaps.

**Objective:**

To examine the mental health status by ethnic group in China and to explore the sociodemographic and health-related factors associated with these mental health disparities.

**Design, Setting, and Participants:**

This cross-sectional study used data from a multicenter, population-based survey conducted from June to August 2023. Participants were required to be aged 18 years or older and Chinese nationals and permanent residents. After quota sampling based on Chinese population statistics, 30 054 questionnaires were included in final analyses. Data were analyzed from March to April 2024.

**Exposures:**

Ethnicity, categorized as Han or minority group (ie, Achang, Bai, Blang, Bonan, Buyei, Dai, Daur, Deang, Derung, Dong, Dongxiang, Ewenki, Gaoshan, Gelao, Gin, Hani, Hezhen, Hui, Jingpo, Jino, Kazak, Kirgiz, Korean, Lahu, Lhoba, Li, Lisu, Man, Maonan, Miao, Monba, Mongol, Mulao, Naxi, Nu, Oroqen, Pumi, Qiang, Russ, Salar, She, Sui, Tajik, Tatar, Tibetan, Tu, Tujia, Uyghur, Uzbek, Va, Xibe, Yao, Yi, Yugur, and Zhuang), as well as unrecognized ethnic groups in China, ie, populations in the People’s Republic of China that have been assimilated into the Han or other recognized ethnic groups but have not yet been officially identified).

**Main Outcomes and Measures:**

Depression and anxiety symptoms were assessed using the Patient Health Questionnaire-9 and Generalized Anxiety Disorder–7-item scale. Any positive (≥1) response to the ninth item of Patient Health Questionnaire-9 was considered as having suicidal ideation.

**Results:**

Among the 30 054 eligible participants (median [IQR] age, 43 [29-54] years; 15 043 [50.1%] female), 27 299 (90.8%) were from the Han ethnic group and 2755 (9.2%) were from ethnic minority groups. Compared with Han participants, participants from ethnic minority groups had significantly higher prevalence of moderate to major depression symptoms (25.5% vs 19.0%; *P* < .001), moderate to severe anxiety symptoms (17.4% vs 12.3%; *P* < .001), and suicidal ideation (29.4% vs 20.9%; *P* < .001). Participants from ethnic minority groups had higher odds of these outcomes even after adjusting for sociodemographic and behavioral factors (moderate to major depression symptoms: adjusted odds ratio [aOR], 1.18; 95% CI, 1.07-1.29; moderate to severe anxiety symptoms: aOR, 1.23; 95% CI, 1.10-1.37; suicidal ideation: aOR, 1.34; 95% CI, 1.22-1.46]). For members of ethnic minority groups, having 2 or more chronic conditions was associated with a higher likelihood of moderate to severe anxiety symptoms (aOR, 1.57; 95% CI, 1.13-2.18) and suicidal ideation (aOR, 1.73; 95% CI, 1.30-2.29).

**Conclusions and Relevance:**

In this cross-sectional study in China, significant disparities of mental health were observed between Han and ethnic minority populations. Members of ethnic minority groups were at a significantly higher risk for experiencing moderate to major depression symptoms, moderate to severe anxiety, and suicidal ideation, particularly when exposed to specific risk factors, such as chronic health conditions and social stressors. Targeted public health interventions are needed to address the mental health needs of ethnic minority populations.

## Key Points

**Question:**

Are there mental health disparities between Han and ethnic minority groups in China, and what sociodemographic and health-related factors are associated with these disparities?

**Findings:**

This cross-sectional study of 30 054 individuals found higher prevalence of moderate to major depression, moderate to severe anxiety, and suicidal ideation among members of ethnic minority groups than Han individuals. After adjusting for sociodemographic and behavioral factors, members of ethnic minority groups had significantly higher odds of experiencing these outcomes.

**Meaning:**

These findings suggest that targeted public health interventions are needed for members of ethnic minority groups in China.

## Teaser

This cross-sectional study examines mental health status by ethnic group in China and explores sociodemographic and health-related factors associated with these mental health disparities.

## Introduction

Globally, racial and ethnic minority populations often experience worse social and health conditions than nonminority populations,^1,2^ and ethnic disparities in mental disorders have been extensively explored.^3,4,5^ Many factors, like employment, income, education, health care policies, social structures, health behaviors, and cultural norms, are believed to contribute to the mental health disparities between groups.^6,7,8,9,10,11,12^

China has one of the largest ethnic minority populations in the world, including 55 ethnic minority groups officially recognized by the Chinese government (Achang, Bai, Blang, Bonan, Buyei, Dai, Daur, Deang, Derung, Dong, Dongxiang, Ewenki, Gaoshan, Gelao, Gin, Hani, Hezhen, Hui, Jingpo, Jino, Kazak, Kirgiz, Korean, Lahu, Lhoba, Li, Lisu, Man, Maonan, Miao, Monba, Mongol, Mulao, Naxi, Nu, Oroqen, Pumi, Qiang, Russ, Salar, She, Sui, Tajik, Tatar, Tibetan, Tu, Tujia, Uyghur, Uzbek, Va, Xibe, Yao, Yi, Yugur, and Zhuang), as well as unrecognized ethnic groups in China, ie, populations in the People’s Republic of China that have been assimilated into the Han or other recognized ethnic groups but have not yet been officially identified. The 2023 census estimated that members of ethnic minority groups account for up to 125 million people, representing 8.9% of the total population.^13^ However, there remain a significant gaps in understanding the mental health of this large population. Current mental health research in China primarily focuses on the Han ethnic majority, leaving a scarcity of data on ethnic minority populations. Previous surveys on China’s ethnic minority groups have primarily focused on specific groups, like Manchu and Tibetan, or specific regions, like western China, reporting depression rates of 2.0% to 35.5% and anxiety rates of 2.2% to 35.1%.^14,15,16,17,18^ In China, the shortage of mental health professionals, weak infrastructure, low population density in many areas, and complex migration patterns make it difficult to provide services to the remote areas inhabited by many members of ethnic minority groups.^19,20^ Understanding the mental health needs of members of ethnic minority groups is critical for developing targeted public health strategies to prevent mental disorders and improve treatment. Therefore, further research is needed to characterize ethnic disparities in mental health outcomes using nationally representative data from China. However, to our knowledge, no published studies have comprehensively examined ethnic disparities in mental health in China, leaving significant gaps in our understanding.

Over the past several decades, the Chinese government has been making significant efforts to improve the rights and opportunities of ethnic minority groups. These measures include political autonomy; economic development through the Western Development Strategy and poverty alleviation; educational equality, with benefits like bonus points in college entrance examinations and special funds; more lenient family planning rules; and cultural preservation policies.^21,22^ However, it is unclear whether these policies improve mental health outcomes among members of ethnic minority groups. This study aims to identify the differences in depressive symptoms, anxiety symptoms, and suicidal ideation between Han individuals and members of ethnic minority groups in a nationally representative sample of China and to explore the sociodemographic and health-related factors contributing to these disparities.

## Methods

This cross-sectional study was approved by the Shandong Provincial Hospital Ethics Review Board and was registered in the Chinese Clinical Trial Registry (registration No. ChiCTR2300072573). Written informed consent was obtained from all participants prior to their participation. This study followed the Strengthening the Reporting of Observational Studies in Epidemiology (STROBE) reporting guideline.

### Study Setting and Population

This study used data from a population-based cross-sectional survey, Psychology and Behavior Investigation of Chinese Residents, conducted in China from June to August 2023 using a multistage, stratified, cluster-sampling method. In stage 1, we randomly selected 160 cities or districts proportional to population size (cities from provinces and autonomous regions, districts from municipalities and special administrative regions). In stage 2, we randomly selected 3 urban communities and 2 rural villages from each city or district. In stage 3, we recruited and screened potentially eligible individuals (60 participants in each community or village) on the street. In stage 4, we adopted postsurvey quota sampling, set on age (5 years interval) and sex, approximating China’s Seventh National Census, to resolve high proportion of younger people because of street-based sample.^20^ A total of 30 054 participants remained and were included in the final analyses. (eFigure 1 in Supplement 1)

The inclusion criteria were aged 18 years or older, Chinese nationality, permanent resident of China (with ≤1 month spent outside the country per year), willing to participate in the study and signed informed consent form, able to complete the online questionnaire independently or with the assistance of the investigator, and understanding the meaning of each item on the questionnaire. The exclusion criteria were having a history of schizophrenia, obsessive-compulsive disorder, bipolar disorder, or current psychotic symptoms; having cognitive impairment (eg, Alzheimer disease) that prevents full comprehension of the questionnaire; or participation in other similar surveys or previous involvement in the Psychology and Behavior Investigation of Chinese Residents survey.

### Data Collection

The data were gathered through online electronic questionnaires surveys by using the Wenjuanxing application (Changsha Ranxing Information Technology) and performed via the social media platform WeChat (Tencent). For participants unable to complete electronic questionnaires (eg, due to illiteracy or disabilities), investigators conducted face-to-face interviews in a private environment. Since only the Mandarin questionnaire was used, local interviewers conducted face-to-face surveys in regional dialects for participants who could not read or understand Mandarin. The survey was anonymous, with no identifiable information collected. Data were kept confidential and accessible only to authorized personnel.

The questionnaire included demographical characteristics, body mass index (BMI), self-reported disease status, traumatic exposure in the past year, adverse childhood experiences (ACEs), the Patient Health Questionnaire 9 (PHQ-9), and Generalized Anxiety Symptoms–7-item (GAD-7). Ethnicity was self-reported via online questionnaire as Han or other.

The disease status included hypertension, diabetes, hyperlipidemia, coronary heart disease, stroke, respiratory system diseases, urinary system diseases, digestive system diseases, osteoporosis, arthritis, cancer, and others. Traumatic exposure investigated injuries and negative life experiences within the past year. Injuries included motor vehicle accidents, falls, burns or scalds, suffocation, drowning, poisoning, and sexual assault. Negative life experiences covered events like the death of family members, unemployment, legal disputes, financial difficulties, natural disasters, property loss, and serious illnesses.

We used the ACEs questionnaire from a previous study,^23^ including 3 categories of childhood abuse (emotional, physical, and sexual abuse) and 4 categories of household dysfunction (substance abuse in the household, mental illness in the household, mother treated violently, and incarcerated household member). Respondents were considered to have ACEs if they answered yes to any question in that category. We assessed the translated questionnaire’s content validity through expert consultation, involving multiple rounds of discussion and revision to ensure each item accurately captured the original intent. The ACE questionnaire in this study has Cronbach α = .86.

Depression symptoms were assessed using PHQ-9. Total scores of 5, 10, 15, and 20 indicated mild, moderate, moderately severe, and severe depression symptoms, respectively.^24^ We referred to a score of 10 or greater as moderate to severe depression symptoms (including moderately severe and severe depression symptoms). Any positive (≥1) response to the ninth item of PHQ-9, which evaluated the thoughts of death or self-injury within last 2 weeks, was considered as having suicidal ideation.^25^ The Chinese version of the PHQ-9 has been validated in primary care, with a Cronbach α = .89. The optimal cutoff score of 10 yields a sensitivity of 0.87 and a specificity of 0.81.^26^

GAD-7 was used to evaluate anxiety symptoms. Total scores of 5, 10, and 15 indicated mild, moderate, and severe anxiety, respectively.^27^ Responses were recorded dichotomously based on a cutoff score of 10 for moderate to severe anxiety symptoms. The Chinese version of the GAD-7 was also reliable and valid, with a Cronbach α = .94. A cutoff score of 10 detects GAD with 86.2% sensitivity and 95.5% specificity.^28^

### Statistical Analysis

We calculated the prevalence and 95% CI of major depressive symptoms, moderate to severe anxiety symptoms, and suicidal ideation by ethnicity and further stratified by sex and age group within each ethnic group. Nonnormal continuous variables were presented as median (IQR) and compared using a Mann-Whitney *U* test. χ^2^ tests were used to compare the differences in prevalence rates between groups. Logistic regression models were performed to estimate the independent association of ethnicity with moderate to major depression symptoms, moderate to severe anxiety symptoms, and suicidal ideation, progressively adjusted for demographic factors (age, sex, residence, educational level, occupational status, mean monthly household income per capita, marital status, number of siblings), lifestyle and life experiences factors (BMI, ACE, recent trauma exposure), and comorbidity factors (disease status). Subgroup analyses by ethnic groups were conducted to estimate the independent associations of these risk factors with moderate to major depression symptoms, moderate to severe anxiety symptoms, and suicidal ideation.

All statistical analyses were performed using Stata version 15.0 (StataCorp). In all analyses, a 2-tailed *P* < .05 was considered statistically significant. Data were analyzed from March to April 2024.

## Results

Among 30 054 eligible participants (median [IQR] age, 43 [29-54] years; 15 043 [50.1%] female) included in the final analysis (eFigure in Supplement 1), 27 299 were from the Han ethnic group (90.8%), and 2755 were from ethnic minority groups (9.2%). Moreover, 20 735 participants (69.0%) resided in urban areas. Overall, 7809 participants (26.0%) experienced at least 1 type of ACE before age 18 years, and 12 569 participants (41.8%) reported a traumatic event in the past year. Significant differences were found between Han and ethnic minority group participants. Participants from ethnic minority groups were younger (median [IQR] age, 40 [27-54] years vs 43 [29-54] years; *P* < .001) and more likely to live in urban areas (36.5% vs 30.5%; *P* < .001). A higher proportion of participants from ethnic minority groups had an undergraduate or above level of education (38.3% vs 35.0%; *P* = .002), but lower household income (Table 1). Recent trauma exposure was more common among members of ethnic minority groups (53.5% vs 40.7%; *P* < .001) (Table 1).

**Table 1.** Sociodemographic and Health Characteristics of the Sample by Ethnicity

Characteristics	Participants, No. (%)			*P* value
	Total (n = 30 054)	Ethnic group		
		Han (n = 27 299)	Minority (n = 2755)^a^	
Age, median (IQR), y	43 (29-54)	43 (29-54)	40 (27-54)	<.001
Sex				
Male	15 011 (49.9)	13 653 (50.0)	1358 (49.3)	.47
Female	15 043 (50.1)	13 646 (50.0)	1397 (50.7)	
Residence				
Urban	9319 (31.0)	8314 (30.5)	1005 (36.5)	<.001
Rural	20 735 (69.0)	18 985 (69.5)	1750 (63.5)	
Education level				
≤Junior high	9243 (30.8)	8419 (30.8)	824 (29.9)	.002
Senior high or specialty education	10 197 (33.9)	9322 (34.2)	875 (31.8)	
≥Undergraduate	10 614 (35.3)	9558 (35.0)	1056 (38.3)	
Occupational status				
Employed	18 345 (61.0)	16 747 (61.4)	1598 (58.0)	<.001
Student	4681 (15.6)	4148 (15.2)	533 (19.4)	
Retired	4228 (14.1)	3863 (14.2)	365 (13.3)	
Unemployed	2800 (9.3)	2541 (9.3)	259 (9.4)	
Mean monthly household income per capita, ¥^b^				
≤3000	8781 (29.2)	7774 (28.5)	1007 (36.6)	<.001
3001 ~ 6000	13 428 (44.7)	12 206 (44.7)	1222 (44.4)	
≥6001	7845 (26.1)	7319 (26.8)	526 (19.1)	
Marital status				
Unmarried (single, divorced or widowed)	10 627 (35.4)	17 907 (65.6)	1520 (55.2)	<.001
Married	19 427 (64.6)	9392 (34.4)	1235 (44.8)	
No. of siblings				
0 (only child)	6970 (23.2)	6378 (23.4)	592 (21.5)	.03
≥1 (non-only child)	23 084 (76.8)	20 921 (76.6)	2163 (78.5)	
BMI				
<18.5	3140 (10.5)	2765 (10.1)	375 (13.6)	<.001
18.5-24.9	21 323 (71.0)	19 424 (71.2)	1899 (68.9)	
25.0-29.9	4913 (16.4)	4511 (16.5)	402 (14.6)	
≥30.0	678 (2.3)	599 (2.2)	79 (2.9)	
ACE				
0	22 245 (74.0)	20 447 (74.9)	1798 (65.3)	<.001
1	3505 (11.7)	3144 (11.5)	361 (13.1)	
2	1930 (6.4)	1741 (6.4)	189 (6.9)	
3	1202 (4.0)	1053 (3.9)	149 (5.4)	
≥4	1172 (3.9)	914 (3.35)	258 (9.4)	
Recent trauma exposure^c^				
No	17, 485 (58.2)	16 203 (59.4)	1282 (46.5)	<.001
Yes	12 569 (41.8)	11 096 (40.7)	1473 (53.5)	
Chronic conditions, No.^d^				
None	20 460 (68.1)	18 728 (68.6)	1732 (62.9)	<.001
1	5812 (19.3)	5281 (19.4)	531 (19.3)	
≥2	3782 (12.6)	3290 (12.1)	492 (17.9)	
Depression symptoms				
Minimal (0-4)	14 619 (48.6)	13 536 (49.6)	1083 (39.3)	<.001
Mild (5-9)	9570 (31.8)	8598 (31.5)	972 (35.3)	
Moderate (10-14)	3406 (11.3)	3005 (11.0)	401 (14.6)	
Moderately severe (15-19)	1787 (6.0)	1572 (5.8)	215 (7.8)	
Severe (≥20)	672 (2.2)	588 (2.2)	84 (3.1)	
Anxiety symptoms				
Minimal anxiety (0-4)	17 479 (58.2)	16 112 (59.0)	1367 (49.6)	<.001
Mild anxiety (5-9)	8749 (29.1)	7839 (28.7)	910 (33.0)	
Moderate anxiety (10-14)	3009 (10.0)	2641 (9.7)	368 (13.4)	
Severe anxiety (≥15)	817 (2.7)	707 (2.6)	110 (4.0)	
Suicidal ideation				
No	23 536 (78.3)	21 591 (79.1)	1945 (70.6)	<.001
Yes	6518 (21.7)	5708 (20.9)	810 (29.4)	

Abbreviations: ACE, adverse childhood experience; BMI, body mass index (calculated as weight in kilograms divided by height in meters squared).

^a^ Includes the 55 ethnic minorities officially recognized by the Chinese government (Achang, Bai, Blang, Bonan, Buyei, Dai, Daur, Deang, Derung, Dong, Dongxiang, Ewenki, Gaoshan, Gelao, Gin, Hani, Hezhen, Hui, Jingpo, Jino, Kazak, Kirgiz, Korean, Lahu, Lhoba, Li, Lisu, Man, Maonan, Miao, Monba, Mongol, Mulao, Naxi, Nu, Oroqen, Pumi, Qiang, Russ, Salar, She, Sui, Tajik, Tatar, Tibetan, Tu, Tujia, Uyghur, Uzbek, Va, Xibe, Yao, Yi, Yugur, and Zhuang), as well as unrecognized ethnic groups in China, ie, populations in the People’s Republic of China that have been assimilated into the Han or other recognized ethnic groups but have not yet been officially identified.

^b^ Approximately less than US$410, US$410 to less than US$820, and US$820 or more.

^c^ Recent Trauma Exposure refers to the experience of significant physical injuries or adverse life events within the past year, including motor vehicle accidents, falls, poisoning, sexual assault, as well as negative life events such as the death of family members, unemployment, legal disputes, financial hardships, and serious illness.

^d^ Including hypertension, diabetes, hyperlipidemia, coronary heart disease, stroke, respiratory system diseases, urinary system diseases, digestive system diseases, osteoporosis, arthritis, cancer, and other diseases.

Using a cutoff score of 10 or higher, 5865 participants (19.5%) had a screening result positive for moderate to major depression symptoms, 3826 participants (12.7%) had moderate to severe anxiety symptoms, and 6518 (21.7%) reported suicidal ideation. Compared with Han participants, members of ethnic minority groups had higher prevalence rates of moderate to major depression symptoms (25.5% vs 19.0%; *P* < .001), moderate to severe anxiety symptoms (17.4% vs 12.3%; *P* < .001), and suicidal ideation (29.4% vs 20.9%; *P* < .001).

**Figure 1.**
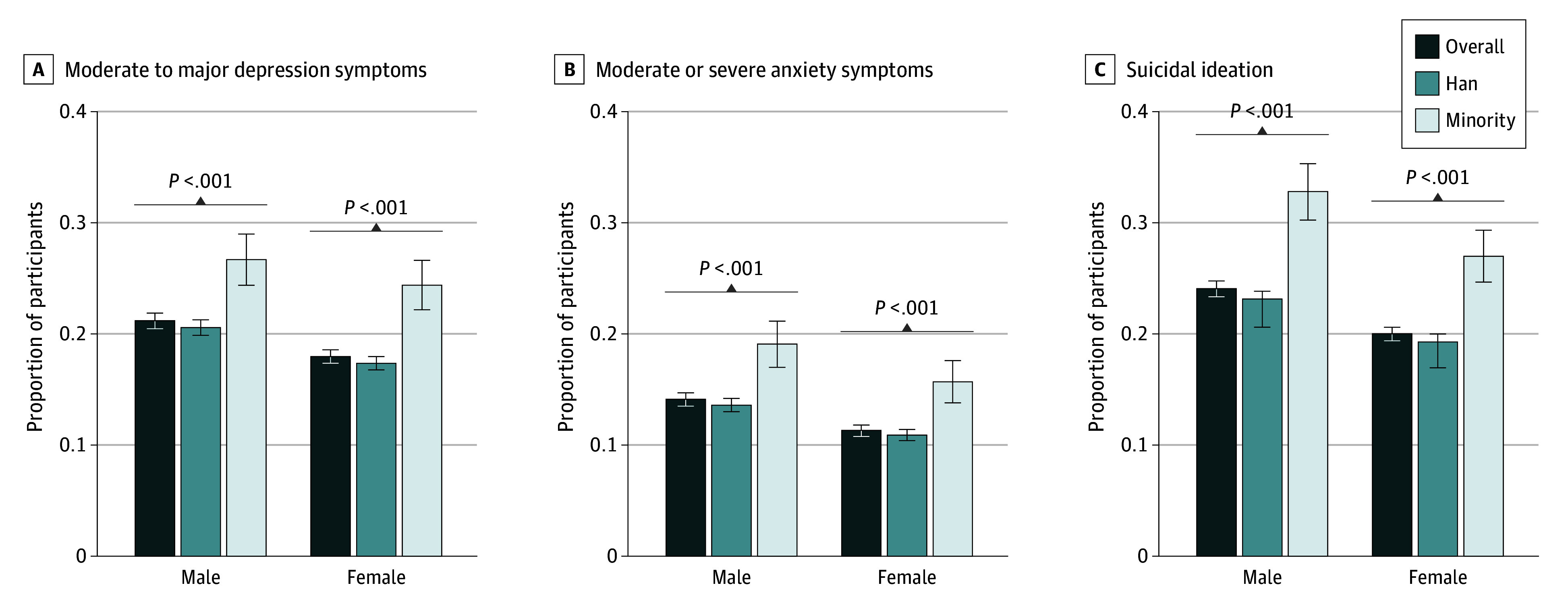
Prevalence of Mental Health Outcomes Among Different Sex and Ethnic Groups The ethnic minority group included Achang, Bai, Blang, Bonan, Buyei, Dai, Daur, Deang, Derung, Dong, Dongxiang, Ewenki, Gaoshan, Gelao, Gin, Hani, Hezhen, Hui, Jingpo, Jino, Kazak, Kirgiz, Korean, Lahu, Lhoba, Li, Lisu, Man, Maonan, Miao, Monba, Mongol, Mulao, Naxi, Nu, Oroqen, Pumi, Qiang, Russ, Salar, She, Sui, Tajik, Tatar, Tibetan, Tu, Tujia, Uyghur, Uzbek, Va, Xibe, Yao, Yi, Yugur, and Zhuang, as well as unrecognized ethnic groups, ie, populations in the People’s Republic of China that have been assimilated into the Han or other recognized ethnic groups but have not yet been officially identified.

**Figure 2.**
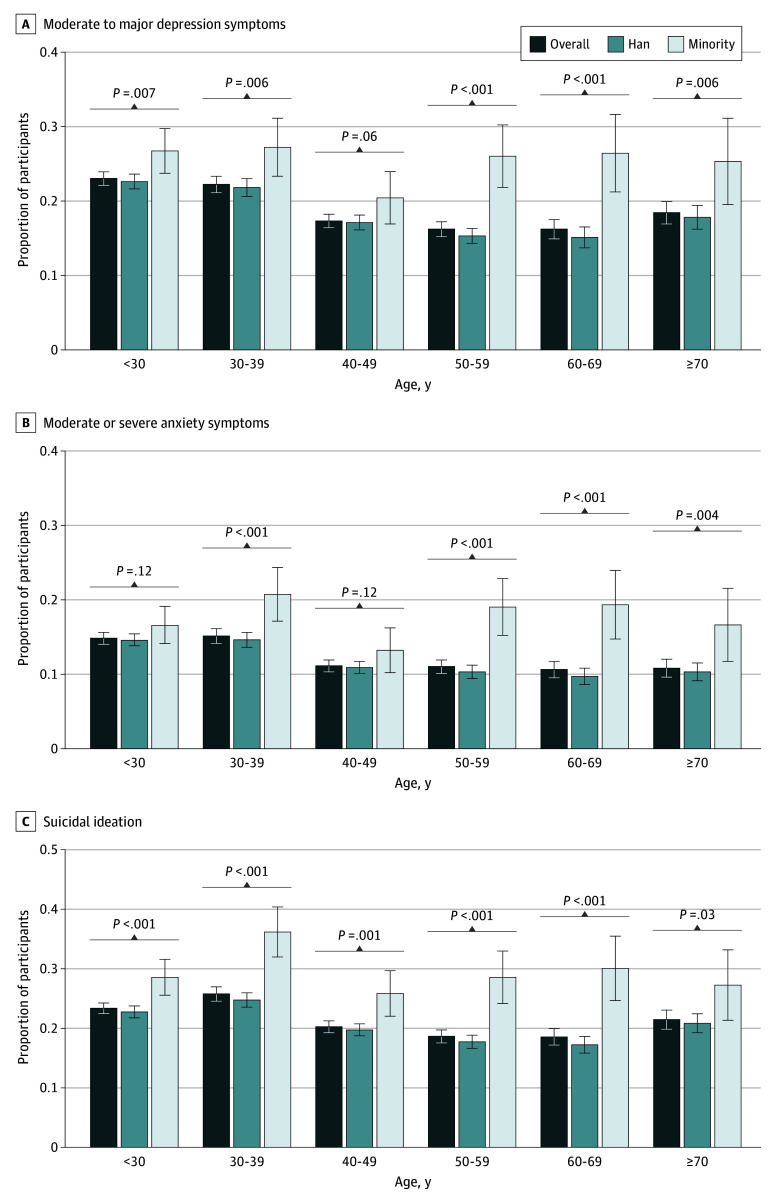
Prevalence of Mental Health Outcomes Among Different Age and Ethnic Groups The ethnic minority group included Achang, Bai, Blang, Bonan, Buyei, Dai, Daur, Deang, Derung, Dong, Dongxiang, Ewenki, Gaoshan, Gelao, Gin, Hani, Hezhen, Hui, Jingpo, Jino, Kazak, Kirgiz, Korean, Lahu, Lhoba, Li, Lisu, Man, Maonan, Miao, Monba, Mongol, Mulao, Naxi, Nu, Oroqen, Pumi, Qiang, Russ, Salar, She, Sui, Tajik, Tatar, Tibetan, Tu, Tujia, Uyghur, Uzbek, Va, Xibe, Yao, Yi, Yugur, and Zhuang, as well as unrecognized ethnic groups, ie, populations in the People’s Republic of China that have been assimilated into the Han or other recognized ethnic groups but have not yet been officially identified.

The sex- and age-stratified analyses demonstrated consistent ethnic disparities in depression symptoms, anxiety symptoms, and suicidal ideation across different subgroups. Members of ethnic minority groups had significantly higher rates of adverse mental health outcomes in both male and female participants (Figure 1). Similarly, ethnic disparities persisted across all age groups, with the exception of the 40 to 49 to age group, in which the differences for moderate to major depression symptoms and moderate to severe anxiety symptoms were not statistically significant (Figure 2). Age-stratified results further indicated that mental health disparities between ethnic groups were more pronounced among participants older than 50 years. In younger age groups, the gap between ethnic minority groups and Han participants narrowed, although younger members of ethnic minority groups still experienced worse outcomes (Figure 2).

Compared with Han participants, participants from ethnic minority groups had higher odds of moderate to major depression symptoms in unadjusted analysis (odds ratio [OR], 1.46; 95% CI, 1.33-1.60), which decreased in adjusted analysis but remained statistically significant (adjusted OR [aOR], 1.18; 95% CI, 1.07-1.29). Similarly, for moderate to severe anxiety symptoms, participants from ethnic minority groups had higher odds in unadjusted analysis (OR, 1.50; 95% CI, 1.35-1.67), which decreased but remained significant in adjusted analysis (aOR, 1.23; 95% CI, 1.10-1.37). The ethnic disparity was most pronounced in odds of suicidal ideation for members of ethnic minority groups (OR, 1.58; 95% CI, 1.44-1.72), and remained high even after adjustment (aOR, 1.34; 95% CI, 1.22-1.46) (Table 2).

**Table 2.** Association of Minority Ethnicity With Moderate to Major Depression Symptoms, Moderate to Severe Anxiety Symptoms, and Suicidal Ideation

Outcome	Odds ratio (95% CI)^a^
**Moderate to major depression symptoms**	
Model 1^b^	1.46 (1.33-1.60)
Model 2^c^	1.37 (1.25-1.50)
Model 3^d^	1.19 (1.08-1.31)
Model 4^e^	1.18 (1.07-1.29)
**Moderate to severe anxiety symptoms**	
Model 1^b^	1.50 (1.35-1.67)
Model 2^c^	1.43 (1.28-1.59)
Model 3^d^	1.25 (1.12-1.39)
Model 4^e^	1.23 (1.10-1.37)
**Suicidal ideation**	
Model 1^b^	1.58 (1.44-1.72)
Model 2^c^	1.51 (1.38-1.65)
Model 3^d^	1.35 (1.23-1.48)
Model 4^e^	1.34 (1.22-1.46)

^a^ For all analyses, Han individuals were the reference group. The ethnic minority group includes the 55 ethnic minorities officially recognized by the Chinese government (Achang, Bai, Blang, Bonan, Buyei, Dai, Daur, Deang, Derung, Dong, Dongxiang, Ewenki, Gaoshan, Gelao, Gin, Hani, Hezhen, Hui, Jingpo, Jino, Kazak, Kirgiz, Korean, Lahu, Lhoba, Li, Lisu, Man, Maonan, Miao, Monba, Mongol, Mulao, Naxi, Nu, Oroqen, Pumi, Qiang, Russ, Salar, She, Sui, Tajik, Tatar, Tibetan, Tu, Tujia, Uyghur, Uzbek, Va, Xibe, Yao, Yi, Yugur, and Zhuang), as well as unrecognized ethnic groups in China, ie, populations in the People’s Republic of China that have been assimilated into the Han or other recognized ethnic groups but have not yet been officially identified.

^b^ Model 1 was analyzed as a univariate model.

^c^ Model 2 was adjusted for demographic factors (age, sex, residence, education level, occupational status, mean monthly household income per capita, marital status, and number of siblings).

^d^ Model 3 adjusted for model 2 factors and lifestyle or behavioral factors (body mass index, adverse childhood experience, and recent trauma exposure).

^e^ Model 4 adjusted for all the factors from models 2 and 3, as well as number of comorbidities.

**Figure 3.**
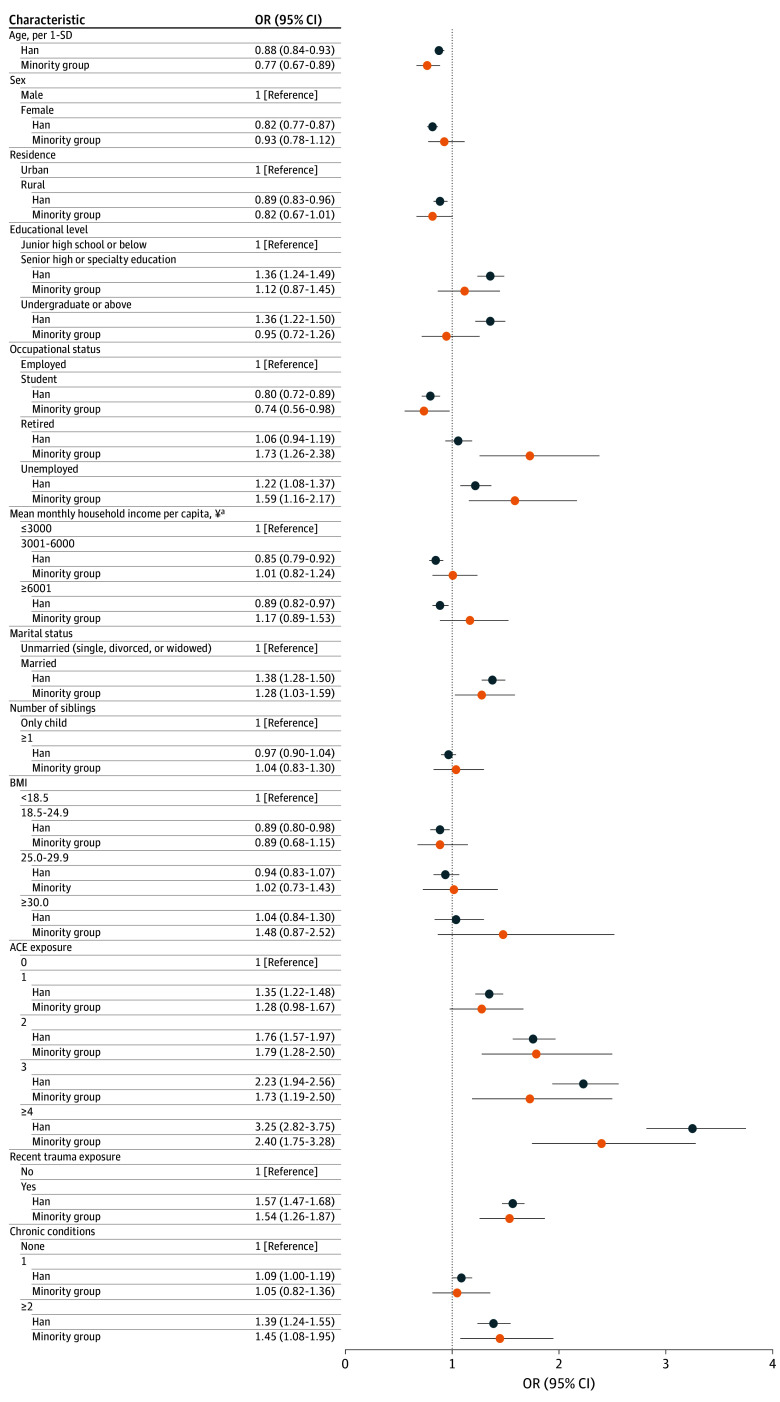
Adjusted Odds Ratios (ORs) for Moderate or Major Depressive Symptoms Between Han and Minority Ethnic Groups Minority ethnic group included Achang, Bai, Blang, Bonan, Buyei, Dai, Daur, Deang, Derung, Dong, Dongxiang, Ewenki, Gaoshan, Gelao, Gin, Hani, Hezhen, Hui, Jingpo, Jino, Kazak, Kirgiz, Korean, Lahu, Lhoba, Li, Lisu, Man, Maonan, Miao, Monba, Mongol, Mulao, Naxi, Nu, Oroqen, Pumi, Qiang, Russ, Salar, She, Sui, Tajik, Tatar, Tibetan, Tu, Tujia, Uyghur, Uzbek, Va, Xibe, Yao, Yi, Yugur, and Zhuang, as well as unrecognized ethnic groups, ie, populations in the People’s Republic of China that have been assimilated into the Han or other recognized ethnic groups but have not yet been officially identified. Data for moderate to severe anxiety symptoms and suicidal ideation are presented in eFigure 2 and eFigure 3 in Supplement 1. ^a^Approximately less than US$410, US$410 to less than US$820, and US$820 or more.

The independent associations of traditional risk factors with moderate to major depression symptoms, moderate to severe anxiety symptoms, and suicidal ideation were estimated using logistic regression models stratified by ethnic group (Figure 3; eFigure 2 and eFigure 3 in Supplement 1). For participants from ethnic minority groups, having 2 or more chronic conditions was associated with higher odds of moderate to severe anxiety symptoms (OR, 1.57; 95% CI, 1.13-2.18) and suicidal ideation (OR, 1.73; 95% CI, 1.30-2.29), although without significant interaction. Similarly, being unemployed showed ethnic differences in the adjusted ORs for these mental health outcomes. Among participants from ethnic minority groups, the odds of being unemployed vs employed were higher for those with moderate to major depression symptoms (OR, 1.59; 95% CI, 1.16-2.17) and moderate to severe anxiety symptoms (OR, 1.56; 95% CI, 1.10-2.22). The interaction between education level and ethnicity was statistically significant in the regression analyses of all 3 mental health outcomes. Among Han participants, having an education level of undergraduate and above was a significant risk factor for all 3 mental health outcomes (moderate to severe depression symptoms: OR, 1.36; 95% CI, 1.22-1.50; moderate to severe anxiety symptoms: OR, 1.22; 95% CI, 1.08-1.38; suicidal ideation: OR, 1.50; 95% CI, 1.36-1.66). However, this association was not observed among participants from ethnic minority groups. Furthermore, the regression model indicated that being exposed to ACEs was associated with more severe depressive symptoms, anxiety symptoms, and suicidal ideation in both Han and ethnic minority groups. However, participants from ethnic minority groups with more ACEs exhibited diminished effect sizes relative to Han participants, although the between-group differences were not statistically significant (Figure 3; eFigure 2 and eFigure 3 in Supplement 1).

## Discussion

The findings of this cross-sectional study highlight significant disparities in mental health outcomes between Han and ethnic minority groups. To our knowledge, this is the first nationwide representative study examining ethnic disparities in mental health in China. Participants from ethnic minority groups exhibited higher prevalence of moderate to major depression symptoms (25.5% vs 19.0%), moderate to severe anxiety symptoms (17.4% vs 12.3%), and suicidal ideation (29.4% vs 20.9%) than Han participants, and these associations remained statistically significant after adjusting for sociodemographic and health factors. Notably, age-stratified analysis showed that these disparities were less pronounced among younger individuals than in those older than 50 years, likely reflecting recent economic development and targeted policies that have improved education and living conditions for ethnic minority youth populations.

Our findings that participants from ethnic minority groups exhibited a higher risk of experiencing moderate to major depression symptoms, moderate to severe anxiety symptoms, and suicidal ideation even after adjusting for confounders suggest that these disparities cannot be fully explained by socioeconomic status or lifestyle factors, echoing studies from the US, Australia, and other regions.^29,30,31^ Potential contributing factors may include cultural, social, and economic stressors that disproportionately affect racial and ethnic minority groups, such as socioeconomic disadvantages and limited access to mental health services.^32,33,34,35,36,37^ Another interpretation for so-called added burden of race refers to the cumulative aggregation of risk factors throughout the life course (eg, variations in the timing, intensity, severity, and duration of exposure to specific risk factors).^38^ Our study also found that ethnic disparities were most pronounced in suicidal ideation, where participants from ethnic minority groups exhibited a significantly higher likelihood of experiencing thoughts of suicide. This underscores the urgent need for targeted suicide prevention strategies within ethnic minority communities. However, suicide ideation screening is uncommon in primary health care settings in China.^39^ Routine suicide risk screening in primary care is recommended to identify individuals at high risk and provide timely support, especially for ethnic minority populations.

Our findings indicated that ethnic minority groups exhibited significantly higher odds of adverse mental health outcomes when exposed to specific risk factors, suggesting that they were more susceptible to certain psychosocial stressors. For instance, having 2 or more diseases was associated with increased risk for moderate to severe anxiety symptoms and suicidal ideation in both groups, but the associations were stronger among participants from ethnic minority groups—possibly due to limited health care access, cultural stigma, and other socioeconomic challenges.^40,41,42,43^ Similarly, unemployment was more strongly associated with moderate to major depression and anxiety symptoms among participants from ethnic minority groups, reflecting additional socioeconomic pressures, such as lower employment opportunities and financial instability.^11,44,45,46^ Furthermore, our data showed that the participants from ethnic minority groups had lower mean income. These results suggest that addressing disparities in health care and employment resources may better support ethnic minority populations in managing mental health disorders.^47,48^

Interestingly, participants from ethnic minority groups exhibited lower sensitivity to certain risk factors. For instance, in regression analyses of our 3 mental health outcomes, an education level of undergraduate and above was not a significant factor for participants from ethnic minority groups, whereas it constituted a risk factor for Han participants. This may stem from the previously mentioned educational policies benefiting ethnic minority populations. Such policies, including affirmative action and targeted support programs, may have contributed to improve resilience resources among individuals from ethnic minority groups, as suggested by previous research.^49^ Moreover, although experiencing 4 or more ACEs was associated with depression and anxiety symptoms, the estimate was significantly lower among participants from ethnic minority groups than Han participants. This may relate to cultural norms, as previous research suggests that individuals in environments with a high prevalence of ACEs might normalize their experiences, which could moderate the negative psychological impact.^50^ This hypothesis is supported by the higher prevalence of ACEs observed among participants from ethnic minority groups in our study.^50^

Since most psychological interventions were developed in North America and Western Europe, they may not fully address the needs of culturally diverse populations. Recent reviews indicate that culturally adapted interventions significantly enhance treatment effectiveness for Chinese populations.^51^ Additionally, studies focusing on ethnic minority groups suggested that culturally adapted interventions yielded better outcomes than nonadapted interventions, and organization-specific modifications may offer further benefits for ethnic minority groups.^52^ Therefore, while developing prevention strategies and treatment plans for mental health issues, it is crucial to consider ethnic and cultural backgrounds and implement culturally adapted interventions.

### Limitations

This study has several limitations. First, due to challenges in accessing participants from extremely remote mountainous areas, our estimates may be subject to bias; however, given their limited numbers, the overall impact on the study findings is expected to be minimal. Second, we collected only binary category information on ethnicity (Han or other ethnicities), which precluded separate analyses for each specific ethnic minority group, thereby potentially masking the heterogeneity in mental health. Third, we did not assess potential confounders, such as discrimination or barriers to accessing mental health care, which may partly explain the higher risk among ethnic minority groups. Fourth, data were collected solely during summer (June to August 2023), without accounting for seasonal or regional variations. Fifth, as the study only adopted a Mandarin-language questionnaire, there may be potential reporting bias for sensitive information among participants who could not read or understand Mandarin, despite the use of face-to-face surveys conducted in regional dialects by local interviewers. Addressing these limitations in future research could significantly enhance the robustness and representativeness of the study findings.

## Conclusions

This cross-sectional study identified significant mental health disparities between Han and ethnic minority populations in China, with ethnic minority groups showing significantly higher prevalence of moderate to major depression, anxiety, and suicidal ideation. These disparities persisted after adjusting for sociodemographic and health factors, highlighting an intrinsic vulnerability. The findings aligned with research from other countries, emphasizing the need for culturally adapted psychological interventions. Targeted strategies to reduce disparities in health care and employment are essential to support racial and ethnic minority populations. Implementing routine suicide risk screening in primary care is recommended, particularly for racial and ethnic minority populations.

## Notes

Supplement 1. **eFigure 1.** Study Flow Diagram **eFigure 2.** Adjusted Odds Ratios for Moderate or Severe Anxiety Symptoms Between Han and Minority Ethnic Groups **eFigure 3.** Adjusted Odds Ratios for Suicidal Ideation Between Han and Minority Ethnic Groups

Supplement 2.
Data Sharing Statement
