# Supplementary material for: Ethnic Disparities in Mental Health Among Adults in China
Source: JAMA Netw Open. 2025 May 15;8(5):e259591. doi: 10.1001/jamanetworkopen.2025.9591 (PMC12082376; doi:10.1001/jamanetworkopen.2025.9591)
Supplement: Supplement 1. — eFigure 1. Study Flow Diagram eFigure 2. Adjusted Odds Ratios for Moderate or Severe Anxiety Symptoms Between Han and Minority Ethnic Groups eFigure 3. Adjusted Odds Ratios for Suicidal Ideation Between Han and Minority Ethnic Groups [file jamanetwopen-e259591-s001.pdf]

Supplemental Online Content

Guo Y, Wu Y, Liu Z, Fan S, Wang H. Ethnic disparities in mental health among adults in China. *JAMA Netw Open*. 2025;8(5):e259591. doi:10.1001/jamanetworkopen.2025.9591

eFigure 1. Study Flow Diagram
eFigure 2. Adjusted Odds Ratios for Moderate or Severe Anxiety Symptoms Between Han and Minority Ethnic Groups
eFigure 3. Adjusted Odds Ratios for Suicidal Ideation Between Han and Minority Ethnic Groups

This supplemental material has been provided by the authors to give readers additional information about their work.

© 2025 Guo Y et al. *JAMA Network Open*.

eFigure 1. Study Flow Diagram

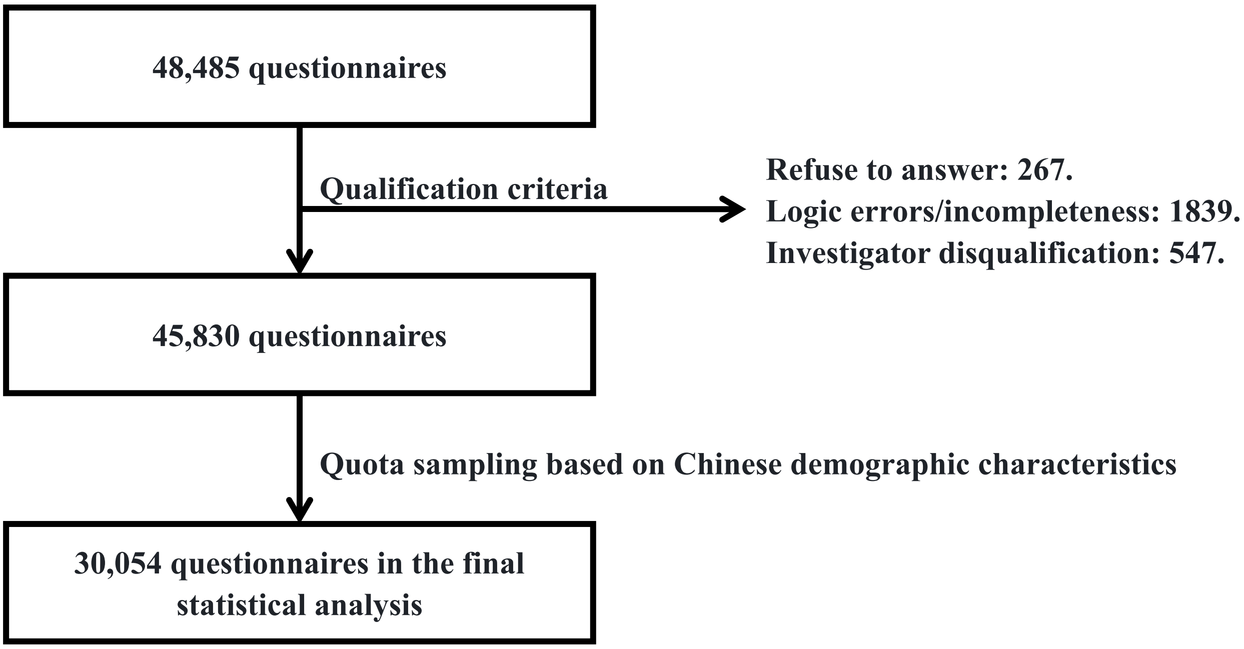

© 2025 Guo Y et al. *JAMA Network Open*.

eFigure 2. Adjusted Odds Ratios for Moderate or Severe Anxiety Symptoms Between Han and Minority Groups

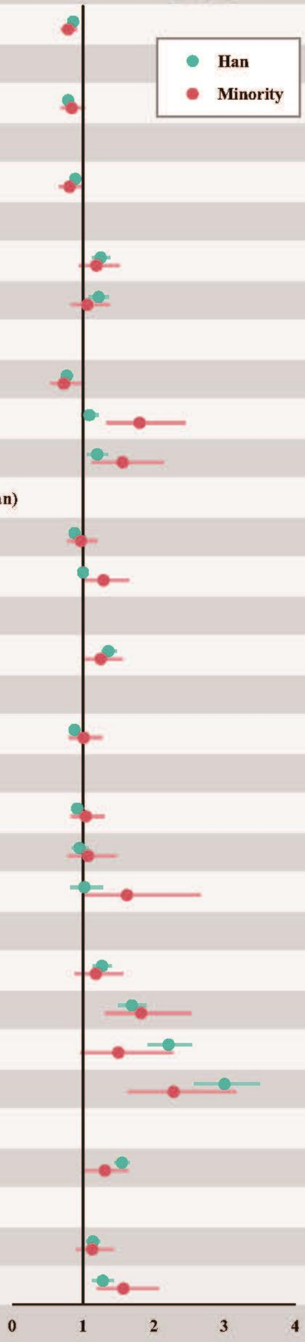

	Moderate or Severe Anxiety Symptoms	OR1	OR2
**Per SD of age (year)**		0.86 (0.81-0.92)	0.79 (0.68-0.92)
**Sex (Ref: male)**		-	-
Female		0.79 (0.73-0.85)	0.84 (0.68-1.03)
**Residence (Ref: Urban)**		-	-
Rural		0.89 (0.82-0.97)	0.81 (0.65-1.03)
**Educational level (Ref: Junior high or below)**		-	-
Senior high or specialty education		1.25 (1.12-1.39)	1.19 (0.89-1.58)
Undergraduate or above		1.22 (1.08-1.38)	1.06 (0.77-1.46)
**Occupational status (Ref: Employed)**		-	-
Student		0.77 (0.68-0.87)	0.73 (0.53-1.01)
Retired		1.09 (0.94-1.26)	1.80 (1.27-2.56)
Unemployed		1.20 (1.05-1.37)	1.56 (1.10-2.22)
**Average monthly household income per capita (Ref: ≤3000 yuan)**		-	-
3001~6000 yuan		0.88 (0.80-0.97)	0.97 (0.76-1.23)
≥6001 yuan		1.00 (0.90-1.11)	1.29 (0.96-1.75)
**Marital status [Ref: Unmarried (single, divorced or widowed)]**		-	-
Married		1.36 (1.24-1.50)	1.25 (0.97-1.60)
**Number of Siblings [Ref: 0 (Only child)]**		-	-
≥1 (Non-only child)		0.88 (0.81-0.96)	1.01 (0.78-1.30)
**BMI [Ref: <18·5 (underweight)]**		-	-
18·5-24·9 (normal)		0.92 (0.82-1.04)	1.04 (0.76-1.41)
25·0-29·9 (overweight)		0.95 (0.82-1.10)	1.07 (0.72-1.59)
≥30·0 (obese)		1.02 (0.79-1.32)	1.62 (0.91-2.91)
**ACE Exposure (Ref: 0 ACE)**		-	-
1 ACE		1.27 (1.14-1.42)	1.18 (0.86-1.61)
2 ACEs		1.69 (1.48-1.93)	1.82 (1.26-2.63)
3 ACEs		2.21 (1.89-2.58)	1.50 (0.99-2.30)
4 or more ACEs		3.00 (2.56-3.50)	2.28 (1.62-3.21)
**Recent Trauma Exposure (Ref: No)**		-	-
Yes		1.55 (1.43-1.68)	1.31 (1.05-1.65)
**Disease Status (Ref: No Chronic Conditions)**		-	-
One Chronic Condition		1.14 (1.03-1.26)	1.13 (0.85-1.51)
Two or More Chronic Conditions		1.28 (1.13-1.46)	1.57 (1.13-2.18)

© 2025 Guo Y et al. *JAMA Network Open*.

eFigure 3. Adjusted Odds Ratios Differences for Suicidal Ideation Between Han and Minority Groups

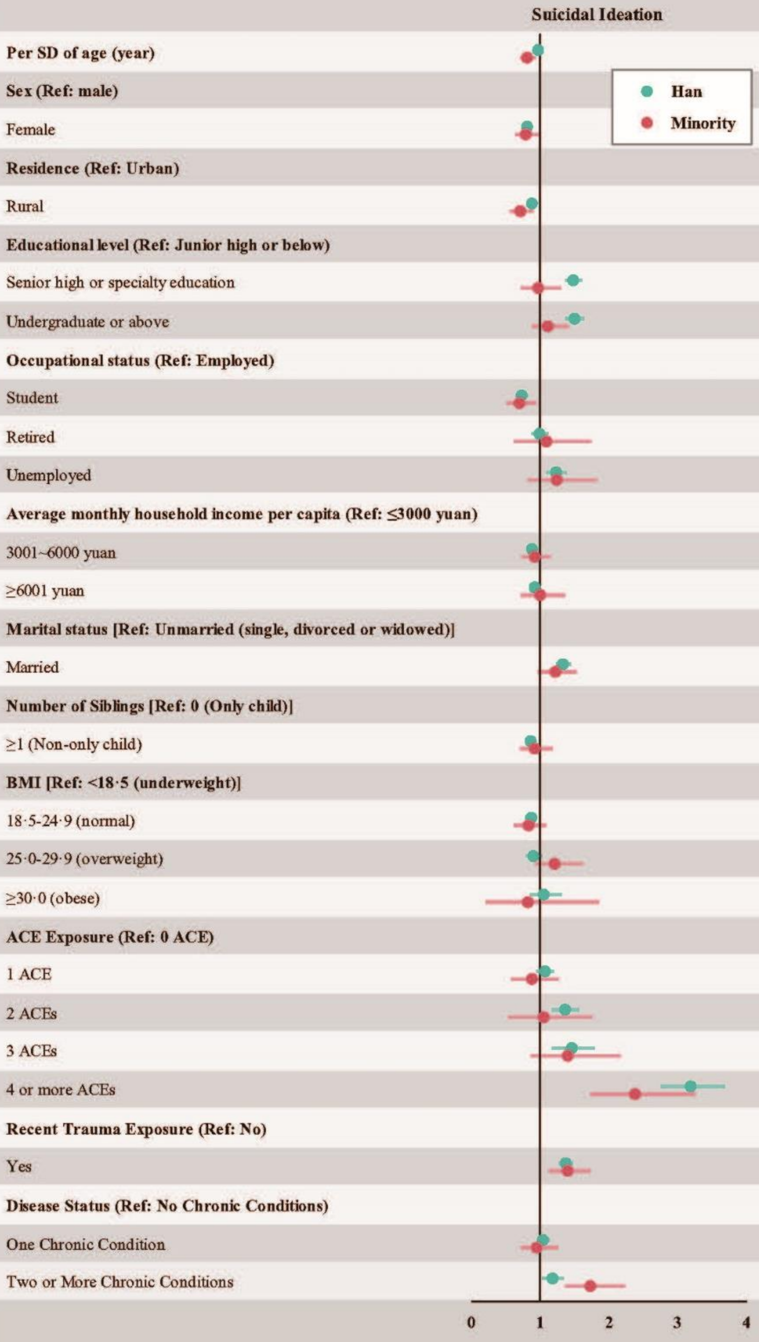

	OR1	OR2
**Per SD of age (year)**	0.97 (0.92-1.03)	0.81 (0.71-0.93)
**Sex (Ref: male)**	-	-
Female	0.81 (0.76-0.86)	0.79 (0.67-0.94)
**Residence (Ref: Urban)**	-	-
Rural	0.88 (0.82-0.94)	0.71 (0.58-0.86)
**Educational level (Ref: Junior high or below)**	-	-
Senior high or specialty education	1.48 (1.36-1.62)	0.97 (0.76-1.24)
Undergraduate or above	1.50 (1.36-1.66)	1.11 (0.85-1.44)
**Occupational status (Ref: Employed)**	-	-
Student	0.73 (0.66-0.81)	0.70 (0.54-0.92)
Retired	0.99 (0.88-1.11)	1.09 (0.79-1.49)
Unemployed	1.23 (1.10-1.38)	1.24 (0.92-1.68)
**Average monthly household income per capita (Ref: ≤3000 yuan)**	-	-
3001~6000 yuan	0.88 (0.82-0.95)	0.92 (0.75-1.12)
≥6001 yuan	0.92 (0.84-1.00)	1.00 (0.77-1.30)
**Marital status [Ref: Unmarried (single, divorced or widowed)]**	-	-
Married	1.33 (1.23-1.43)	1.22 (0.98-1.51)
**Number of Siblings [Ref: 0 (Only child)]**	-	-
≥1 (Non-only child)	0.86 (0.80-0.93)	0.92 (0.75-1.14)
**BMI [Ref: <18·5 (underweight)]**	-	-
18·5-24·9 (normal)	0.87 (0.78-0.95)	0.83 (0.64-1.07)
25·0-29·9 (overweight)	0.90 (0.80-1.01)	1.21 (0.88-1.67)
≥30·0 (obese)	1.05 (0.85-1.29)	0.82 (0.47-1.42)
**ACE Exposure (Ref: 0 ACE)**	-	-
1 ACE	1.07 (0.98-1.18)	0.88 (0.67-1.15)
2 ACEs	1.36 (1.22-1.53)	1.05 (0.75-1.49)
3 ACEs	1.46 (1.26-1.68)	1.40 (0.97-2.01)
4 or more ACEs	3.19 (2.77-3.68)	2.38 (1.75-3.23)
**Recent Trauma Exposure (Ref: No)**	-	-
Yes	1.37 (1.28-1.46)	1.40 (1.16-1.69)
**Disease Status (Ref: No Chronic Conditions)**	-	-
One Chronic Condition	1.04 (0.96-1.13)	0.95 (0.74-1.22)
Two or More Chronic Conditions	1.18 (1.06-1.32)	1.73 (1.30-2.29)

© 2025 Guo Y et al. *JAMA Network Open*.
