# Supplementary material for: Ethnic Disparities in Mental Health Among Adults in China
Source: JAMA Netw Open. 2025 May 15;8(5):e259591. doi: 10.1001/jamanetworkopen.2025.9591 (PMC12082376; doi:10.1001/jamanetworkopen.2025.9591)
Supplement: Supplement 2. — Data Sharing Statement [file jamanetwopen-e259591-s002.pdf]

Data Sharing Statement

Guo. Ethnic Disparities in Mental Health Among Adults in China. *JAMA Netw Open*. Published May 15, 2025. doi:10.1001/jamanetworkopen.2025.9591

Data
Data available: Yes
Data types: Deidentified participant data
How to access data: bjmuwuyibo@outlook.com
When available: With publication

Supporting Documents
Document types: None

Additional Information
Who can access the data: researchers whose proposed use of the data has been approved
Types of analyses: for any purpose or for a specified purpose
Mechanisms of data availability: with a signed data access agreement
